# Supplementary material for: Development of an LC-MS/MS Method for ARV-110, a PROTAC Molecule, and Applications to Pharmacokinetic Studies
Source: Molecules. 2022 Mar 18;27(6):1977. doi: 10.3390/molecules27061977 (PMC8954769; doi:10.3390/molecules27061977)
Supplement: Supplementary file 1 [file molecules-27-01977-s001.zip › molecules-1617203-suppl.pdf]

molecules

MDPI

*Supplementary Materials*

# Development of an LC-MS/MS method for ARV-110, a PROTAC molecule, and applications to pharmacokinetic studies

**Thi-Thao-Linh Nguyen[1,#], Jin Woo Kim[2,#], Hae-in Choi[2], Han-Joo Maeng[1,*], Tae-Sung Koo[2,*]**

[1] College of Pharmacy, Gachon University, 191 Hambakmoe-ro, Yeonsu-gu, Incheon 21936, Republic of Korea
[2] Graduate School of New Drug Discovery and Development, Chungnam National University, Daejeon 34134, Republic of Korea
# These authors contributed equally to this work
* Correspondence: hjmaeng@gachon.ac.kr (H.-J.M.); kootae@cnu.ac.kr (T.-S.K.); Tel.: +82-32-820-4935 (H.-J.M.); +82-42-821-8628 (T.-S.K.)

## ARV-110: $^1$H-NMR (400 MHz, $CDCl_3$)

$^1$H NMR (400 MHz, $CDCl_3$) δ 7.97 (d, *J* = 9.6 Hz, 1H), 7.95 (s, 1H), 7.87 (d, *J* = 8.2 Hz, 1H), 7.56 (d, *J* = 8.7 Hz, 1H), 7.48 (d, *J* = 11.1 Hz, 1H), 7.38 (d, *J* = 7.3 Hz, 1H), 7.01 (s, 1H), 6.98 (d, *J* = 9.6 Hz, 1H), 6.86 (dd, *J* = 8.7, 2.4 Hz, 1H), 4.94 (dd, *J* = 12.3, 5.3 Hz, 1H), 4.52 (d, *J* = 13.2 Hz, 2H), 4.38 – 4.29 (m, 1H), 4.10 – 4.00 (m, 1H), 3.34 – 3.24 (m, 4H), 3.12 – 3.00 (m, 2H), 2.95 – 2.69 (m, 3H), 2.65 – 2.58 (m, 4H), 2.30 (d, *J* = 6.9 Hz, 2H), 2.24 – 2.09 (m, 5H), 2.00 – 1.93 (m, 2H), 1.92 – 1.85 (m, 1H), 1.75 – 1.64 (m, 2H), 1.51 – 1.42 (m, 2H), 1.32 – 1.25 (m, 2H).

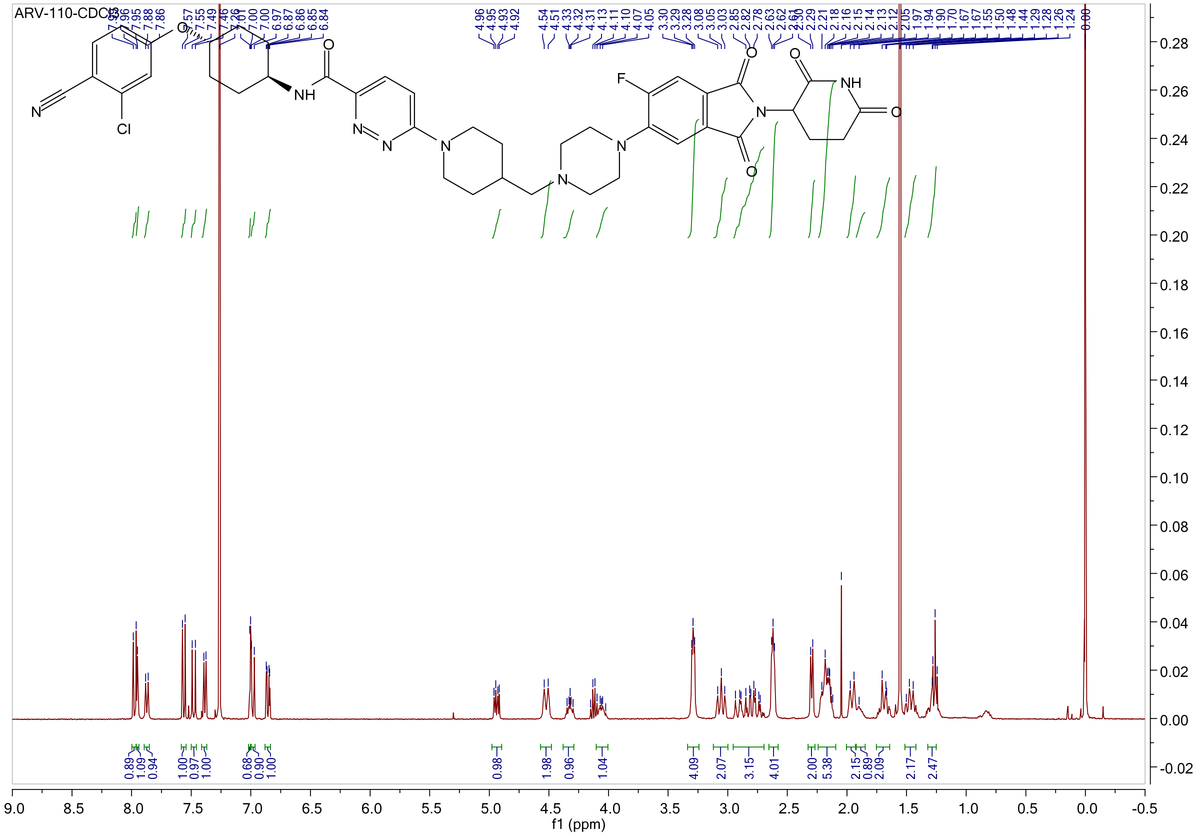


**Figure S1.** $^1$H-NMR (400 MHz, $CDCl_3$) spectra of ARV-110

## ARV-110: Mass

HPLC-QDA/ESI-MS (Waters) equipped with Empower 3.0 software
m/z 812.26 $[M+H]^+$.

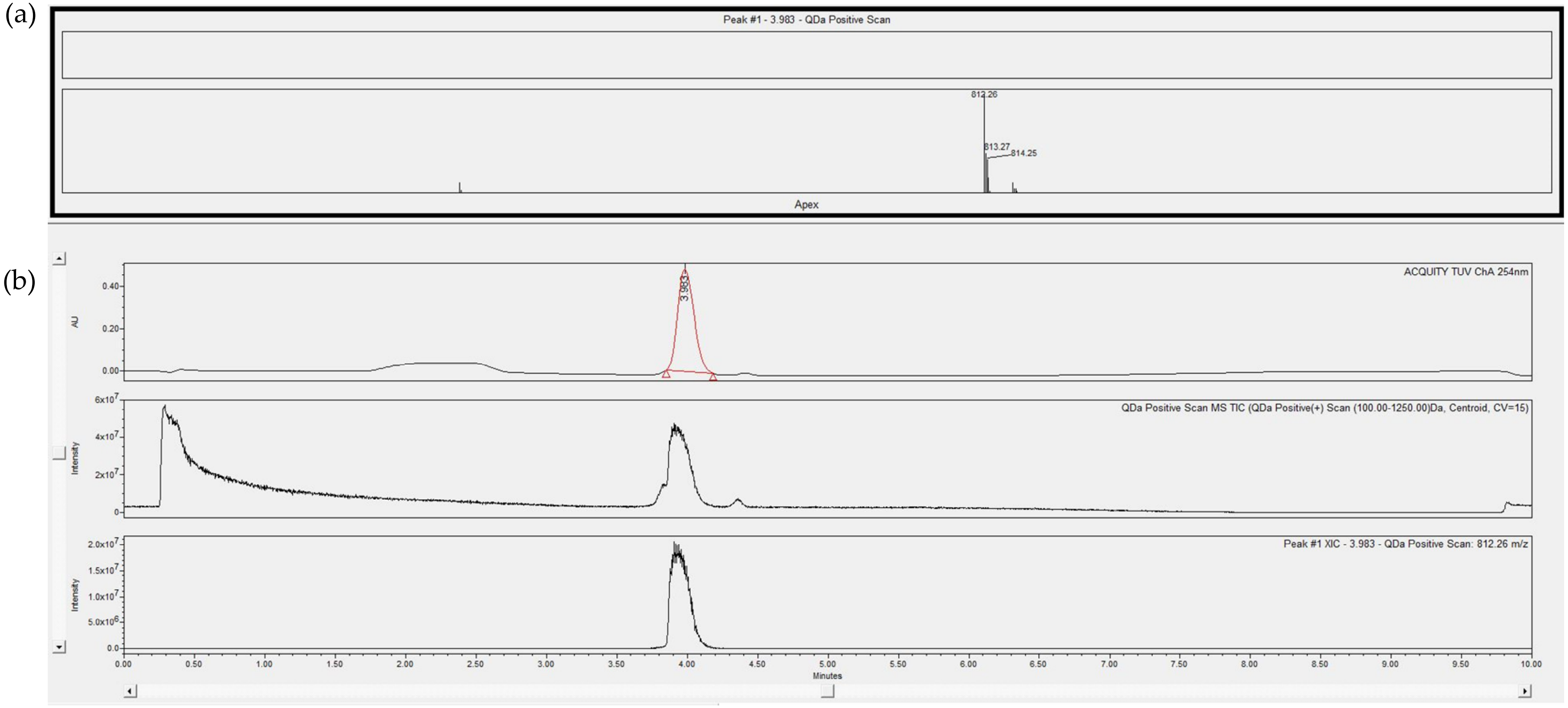


**Figure S2.** QDa-MS profile of ARV-110 (**a**) and high-performance liquid chromatography-single quadrupole mass spectrometry (HPLC-UV-QDa) chromatograms for ARV-110 (**b**).
